# Benefits of prophylactic voice prosthesis replacement: a retrospective study

**DOI:** 10.3389/fonc.2025.1566697

**Published:** 2025-06-25

**Authors:** Sławomir Okła, Jakub Spałek, Szczepan Kaliniak, Agnieszka Strzelecka, Michał Chrobot, Paweł Macek, Michiel W. van den Brekel, Stanisław Góźdź

**Affiliations:** ^1^ Institute of Medical Science, Collegium Medicum, Jan Kochanowski University of Kielce, Kielce, Poland; ^2^ Department of Otolaryngology, Head and Neck Surgery, Holy-Cross Cancer Center, Kielce, Poland; ^3^ Institute of Health Science, Collegium Medicum, Jan Kochanowski University of Kielce, Kielce, Poland; ^4^ Scientific Research, Epidemiology and R&D Centre, Holy-Cross Cancer Centre, Kielce, Poland; ^5^ Department of Head and Neck Oncology and Surgery, The Netherlands Cancer Institute, Amsterdam, Netherlands

**Keywords:** voice prosthesis (VP), prophylactic VP replacement, TE fistula widening, device lifetime, complication, laryngectomy, biofilm formation

## Abstract

**Introduction:**

In this retrospective, single-center study conducted at a regional cancer center, we have analyzed whether prophylactic voice prosthesis replacement (PVPR) could reduce the occurrence of tracheoesophageal fistula (TEF) dysfunction.

**Methods:**

We reviewed 2,431 cases of voice prosthesis (VP) replacement procedures performed in 327 patients between January 2017 and December 2022 at the Department of Otolaryngology, Head and Neck Surgery, Holy Cross Cancer Centre, Kielce, Poland. In the middle of this period (January 2020), the management of VP replacements was changed from reactive, unscheduled voice prosthesis replacement (UVPR), with a median device lifetime of 7 months, to prophylactic, scheduled replacement (PVPR) procedures occurring every 3 months.

**Results:**

The statistical analysis confirmed a significantly lower number of complications during the period of PVPR (2020–2022) compared to the previous period of UVPR (2017–2019). In the years 2017–2019, out of a total of 911 voice prosthesis replacements performed in 246 patients, 425 were associated with complications related to TEF (47%). In comparison, in the years 2020–2022 (following the introduction of PVPR), only 91 cases (6%) (p<0.001; r = 0.408) out of 1,520 voice prosthesis exchanges performed in 250 patients had related TEF complications. The types and occurrence of TEF complications remained the same in both time intervals (UVPR vs. PVPR), with widening of the fistula tract being the most common, comprising 80% and 78% of all TEF complications, respectively.

**Discussion:**

PVPR every 3 months can reduce fistula complications compared to a protocol with reactive replacement for voice prosthesis or TEF dysfunction.

## 1 Introduction

The estimated global numbers of new cases of laryngeal cancer were 184,615 and 84,254 for hypopharyngeal cancer in 2020 (1). Patients with advanced locoregional disease, after failure of organ-preservation treatment, are advised to undergo a total laryngectomy, which leads to loss of a normal laryngeal voice. The most effective method of voice restoration is tracheoesophageal puncture (TEP) with voice prosthesis (VP) placement that can be easily performed, primarily during total laryngectomy or secondarily as a delayed procedure (2–6). Regardless of all the benefits, this method also has some disadvantages. Uncontrolled leakage around the VP has been considered a main concern since the introduction of surgical voice rehabilitation with the insertion of a VP by M. Singer and E. Bloom in 1978 (7). Chronic aspiration, often clinically asymptomatic, can still lead to recurrent pneumonia, with the potential to be life-threatening to patients (8, 9). The voice prostheses are made of a silicone polymer, which is susceptible to colonization and deformation by bacteria and fungi. The process of biofilm growth is the main driver of VP damage and deformation, which leads to its dysfunction (10–13). Apart from transprosthetic leakage, which is the leading cause of VP replacement, complications related to the TE fistula remain the most problematic and threatening. Inflammation, edema, hypertrophy, and widening of the TE fistula can often be solved through spontaneous healing and/or shrinking following the removal of the VP. Patients then require a nasogastric tube for feeding and sometimes a temporary cuffed cannula to prevent aspiration. Additional conservative treatment, such as antibiotics, antifungal drugs, proton pump inhibitors, thyroid hormone supplementation, and balanced nutrition, is also necessary. For persistent fistula enlargements, the next step procedures are hyaluronic acid injections, purse string sutures on the fistula tract, and, as a last resort, surgical closures may be used.

The average voice prosthesis lifetime is variable in different studies and ranges from 2 to more than 10 months (6, 14–28). The Department of Otolaryngology, Head and Neck Surgery, Holy Cross Cancer Centre, Kielce, Poland, started surgical voice rehabilitation using VP in 2001. This was the first center in Poland to routinely begin implanting voice prostheses for voice and speech rehabilitation after total laryngectomy. Fearing the potential complications (8, 9), we adopted the principle of treating tracheoesophageal fistula dysfunction by admitting the patients to the department. This was also due to the National Healthcare System (NHS) requirement to perform voice prosthesis replacement only as a hospital procedure, so that the hospital receives reimbursement. More so, esophageal speech was considered the gold standard of treatment in the rehabilitation of patients after total laryngectomy in many other centers in Poland. Initially, the NHS restrictions allowed only a single VP reimbursement per year, with the median device lifetime (DL) being more than 12 months. Since the removal of this restriction in 2010, the median DL has been slowly decreasing, reaching 7 months in 2017. Following the observation of the progressive growth of biofilm colonies on voice prostheses (13) and the large number of observed fistula problems in this period, we proposed a theory that a longer DL may lead to more fistula problems. This may possibly be in relation to the irritation of local tissues caused by the biofilm. Hence, we set out to investigate the hypothesis that prophylactic voice prosthesis replacement (PVPR) may be beneficial in reducing fistula complications. As such, from January 2020, every patient with a VP was called in for a prophylactic replacement every 3 months.

## 2 Materials and methods

This study was designed as a retrospective cohort, single-center study. All (n = 2,431) voice prosthesis replacement procedures performed in 327 patients at the Department of Otolaryngology, Head and Neck Surgery from January 2017 to December 2022 were included. Between 2017 and 2019, the VP replacements took place as an unscheduled, reactive procedure depending on the VP or fistula dysfunction. From 2020 to 2022, the patients were called in for a scheduled PVPR every 3 months (17). The procedure technique and the type of voice prosthesis remained unchanged, with the only difference being that the VP replacement between 2020 and 2022 was performed by only the most experienced physicians, specifically limited to 6 out of 17 department physicians. The specific VP used was a 22.5 French indwelling VP (ATOS Medical: Provox 2, Provox Vega, Provox Vega XS).

In total, 327 individual patients with voice prostheses after total laryngectomy were included in this study. A primary tracheoesophageal puncture was performed during the TL in 77% of all the studied patients (**Table 1**). During the observation period, some patients died due to disease recurrence or comorbidities, but new patients joined the study group following diagnosis and treatment. Only 84 patients passed through the entire 6-year study period. The number of patients in the groups remained similar each year, ranging from 167 to 196 patients. In this research, 1-year periods were analyzed and described (**Table 2** **).** Then, the single years were combined into two groups, with 2017–2019 comprising the unscheduled voice prosthesis replacement (UVPR) group and 2020–2022 comprising the PVPR group. These two groups (UVPR vs. PVPR) were subjected to statistical analysis to find differences and correlations (**Table 3** **).** A group of 84 patients who were present throughout the entire study period was analyzed separately to compare the two periods (UVPR and PVPR) in a more homogeneous study group.

**Table 1:** Study group information.

Years	2017–2022	2017–2019 UVPR	2020–2022 PVPR	p-value
Sex				0.209
Male	293 (90%)	223 (91%)	220 (88%)	
Female	34 (10%)	23 (9%)	30 (12%)	
All individual patients	327	246	250	
Type of VP implantation				0.831
Primary VP implantation	252 (77%)	188 (76%)	191 (76%)	
Secondary VP implantation	57 (17%)	46 (19%)	44 (18%)	
Laryngectomy and VP implantation in another hospital	18 (6%)	12 (5%)	15 (6%)	
n	327	246	250	
Indication for TL				0.773
Primary TL	28 (11%)	20 (11%)	22 (12%)	
Primary TL + adjuvant RT/CRT	181 (72%)	138 (73%)	134 (70%)	
Salvage TL	43 (17%)	30 (16%)	35 (18%)	
n*	252 (100%)	188 (100%)	191 (100%)	
Neck dissection during TL				0.442
none	39 (15%)	39 (21%)	30 (16%)	
SND unilateral	9 (4%)	8 (4%)	6 (3%)	
SND bilateral	181 (72%)	123 (65%)	140 (73%)	
(m)RND	23 (9%)	18 (10%)	15 (8%)	
n*	252 (100%)	188 (100%)	191 (100%)	
Cancer stage (pathological)				0.923
I	8 (3%)	6 (3%)	8 (4%)	
II	30 (12%)	23 (12%)	26 (14%)	
III	70 (28%)	56 (30%)	54 (28%)	
IV	144 (57%)	103 (55%)	103 (54%)	
n*	252 (100%)	188 (100%)	191 (100%)	
Primary tumor site				0.999
Hypopharynx	34 (13%)	26 (14%)	27 (14%)	
Supraglottic	75 (30%)	56 (30%)	48 (25%)	
Glottic	126 (50%)	93 (49%)	103 (54%)	
Subglottic	17 (7%)	13 (7%)	13 (7%)	
n*	252 (100%)	188 (100%)	191 (100%)	

The chi-square test was used to compare the qualitative variables. No statistically significant relationship was found between the variables studied, p-value test >α 0.05 (χ2 test). TL, total laryngectomy; SND, selective neck dissection; (m)RND, (modified) radical neck dissection; UVPR, unscheduled voice prosthesis replacement; PVPR, prophylactic voice prosthesis replacement. *number of patients include patients with primary voice prosthesis (VP) implantation only.

**Table 2:** The number of voice prosthesis replacement (VPR) procedures performed each year from 2017 to 2022.

Variable	Year					
	2017	2018	2019	2020	2021	2022
Number of patients, n	172	167	182	169	192	196
Number of all VPR procedures, n	315	283	313	329	533	658
Number of VPR procedures without fistula complications, n (%)	185 (59)	118 (42)	183 (59)	280 (85)	503 (94)	646 (98)
Number of VPR procedure with fistula complications: n (%)	130 (41)	165 (58)	130 (41)	49 (15)	30 (6)	12 (2)
Total days of hospitalization due to fistula complications, n	513	663	644	245	133	58
Number of patients with fistula complications n (%)	90 (52)	104 (62)	89 (49)	40 (24)	26 (14)	11 (6)
Average number of VPRs per single patient	1.83	1.69	1.72	1.95	2.78	3.36

Any voice prosthesis replacement associated with an unscheduled hospitalization of more than 1 day was considered a fistula complication. UVPR, unscheduled voice prosthesis replacement; PVPR, prophylactic voice prosthesis replacement.

**Table 3:** The number of voice prosthesis replacement (VPR) procedures performed in the two compared periods.

Variable	UVPR (2017–2019)	PVPR (2020–2022)	2017–2022 (Total)	Median per patient in the UVPR group	Median per patient in the PVPR group
Number of patients	246	250	327	–	–
Number of all VPR procedures	911	1520	2431	3.0	6.0
Number of VPR procedures without fistula complications, n (%)	486 (53)	1429 (94)	1915 (79)	1.0	6.0
Number of VPR procedures with fistula complications, n (%)	425 (47)	91 (6)	516 (21)	1.0	0.0
Total days of hospitalization due to fistula complications	1820	436	2256	5.0	0.0
Total days of hospitalization (all causes)	2731	1956	4687	7.0	7.0
Number of patients with fistula complications, n (%)	113 (54)	35 (14)	–	–	–
Number of patients with hospitalization >14 days, n (%)	61 (25)	27 (11)	–	–	–

UVPR, unscheduled voice prosthesis replacement; PVPR, prophylactic voice prosthesis replacement.

Data for the UVPR (2017–2019) and PVPR (2020–2022) periods are presented separately. The column “2017–2022 (Total)” shows cumulative values for the entire study period. “Median” represents the median number of procedures or hospitalization days per individual patient in each group. Total hospitalization days include both planned and complication-related admissions. The percentage of patients with hospitalization >14 days was calculated using a unified threshold for both groups. VPR, voice prosthesis replacement.

Every time a patient was hospitalized for more than 1 day, the event was scored as a complication. If a patient was hospitalized for only 1 day, it was assumed to be a routine replacement without any complications. All fistula complications were treated with a hospital admission, removal of the prosthesis, a feeding tube, and replacement after shrinkage and/or healing.

### 2.1 Statistical analysis

The statistical analysis was performed to compare outcomes between the UVPR and PVPR groups. The significance level for all statistical tests was set at α=0.05 (p < 0.05).

The Mann–Whitney U test was used to compare the number of voice prosthesis replacement (VPR) procedures and the number of hospitalization days related to complications between the groups. The effect size (r) was calculated using the formula r = Z/√N, where Z represents the standardized statistic and N is the total number of observations. The effect size values were interpreted according to the following standard thresholds: approximately 0.1: small; 0.3: medium; and ≥0.5: large effect. The χ² test was applied to compare the incidence of complications at baseline (UVPR vs. PVPR) and to evaluate the relationship between period (UVPR and PVPR) and the type of complications. The Spearman correlation test was used to assess the relationship between the number of VPR procedures and the incidence of complications per patient. For the dynamic cohort of patients, complication incidence rates and the percentage of complication-free patients were calculated. The incidence rate was expressed as the number of complications per person-year, assuming a 3-year observation period for each patient in both the UVPR and PVPR periods. The percentage of complication-free patients was calculated as the proportion of patients without any complications during each respective period. Logistic regression analysis was also performed to predict the occurrence of prolonged hospitalizations (>1 day). Model performance was assessed using the area under the ROC curve (AUC = 0.812), indicating good predictive ability. The optimal cutoff point was determined using the Youden Index, which maximizes the sum of sensitivity and specificity. The statistical significance of predictors in the logistic regression model was verified using Wald’s test (Wald’s p-value). All statistical analyses were conducted using STATISTICA version 13.1 advanced analytics software.

### 2.2 Institutional review board statement

The study was approved by the Ethics Committee of Jan Kochanowski University of Kielce, with the following protocol code: 26/2023.

## 3 Results

In total, 293 men and 34 women (327 in total) within an age range of 31 to 88 years old [average age of 65; standard deviation (SD) of 8.5] were included in this study. Age was not correlated with the incidence of complications (verification was based on Wald’s p-value). The average time between the VP implantation (primary or secondary) to patient inclusion in this study was 3.32 years (ranged between 2 months to 16 years). **Table 1** presents the clinical characteristics of the patients included in this study. No statistically significant differences were found between the two groups (UVPR vs. PVPR) regarding the type of implantation, the prior treatment received, the location of the primary tumor, or the clinical stage of cancer. The number of patients and procedures in each individual year is shown in **Table 2**. In 2020, the number of VP replacement procedures started to rise gradually. The lowest number of voice prostheses carried out per patient occurred in 2018 (with an average of 1.69); this had almost doubled by 2022 (average 3.36). The median VP replacement per patient was between 1 to 2 during the UVPR period and 3 to 4 in the PVPR period, with the median reaching 4 in 2022. Since the implementation of PVPR in 2020, a marked and consistent reduction was observed not only in the frequency of VP exchanges due to TEF complications and the corresponding hospitalizations, but also in the overall incidence of such complications among patients. Specifically, in 2017, such adverse events were reported in 52% of the patients (90 out of 172 patients), whereas in 2020, this decreased to 24% (40 out of 169 patients). Most recently, in 2022, the incidence further diminished, as only 6% of the patients experienced complications (11 out of 196 patients). **Table 3** presents the number of patients, the total number of VPR procedures performed, and the total number of hospitalization days associated with both complications and all hospital admissions in the UVPR (2017–2019) and PVPR (2020–2022) groups and during the entire follow-up period (2017–2022). Additionally, to provide a more accurate and balanced comparison, the median number of procedures and median number of hospital days per patient in each group were calculated, as was the percentage of patients with hospitalizations lasting more than 14 days. The threshold of 14 days was based on the 75th percentile of the number of hospital days in the UVPR group, representing standard clinical practice before the introduction of PVPR. Using a fixed threshold allows for a fair comparison of the rates of prolonged hospitalizations between the groups. During the PVPR period, significant reductions in the median number of procedures with complications (UVPR: 1.0; PVPR: 0.0) and the median number of hospitalization days related to complications (UVPR: 5.0; PVPR: 0.0) were observed. The results of the Mann–Whitney U test showed that these differences were statistically significant (p < 0.0001) and characterized by a medium to large effect size (r=0.408 for the number of procedures with complications and 0.387 for hospitalization days, respectively). The effect size was large for the procedures without complications (r = 0.530, p < 0.0001), indicating a significantly higher median number of procedures without complications during the PVPR period. We calculated the effect size (r) based on the results of the Mann–Whitney U test using the formula r = Z/√N, where Z is the value of the standardized statistic and N is the total number of observations. The interpretation of the effect size was performed according to commonly accepted thresholds (r ≈ 0.1 = small effect; r ≈ 0.3 = medium effect; r ≥ 0.5 = large effect). The results, including both the effect sizes and p-values, are presented in **Table 4**.

**Table 4:** Comparison of the median number of procedures and hospitalization days between the UVPR and PVPR groups with effect size (r) and p-value.

Variable	Effect size (r)	p-value
Number of VPR with complications	0.408	<0.0001
Number of VPR without complications	0.530	<0.0001
Days of hospitalization due to complications	0.387	<0.0001

Effect size (r) was calculated based on the Mann–Whitney U test, using the formula r = Z/√N, where Z is the Z-score from the Mann–Whitney U test, and N is the total number of observations. Interpretation of r: small effect = 0.1, medium effect = 0.3, large effect ≥ 0.5. Significance level set at α = 0.05.VPR, voice prosthesis replacement; UVPR, unscheduled voice prosthesis replacement; PVPR, prophylactic voice prosthesis replacement.

**Figure 1:**
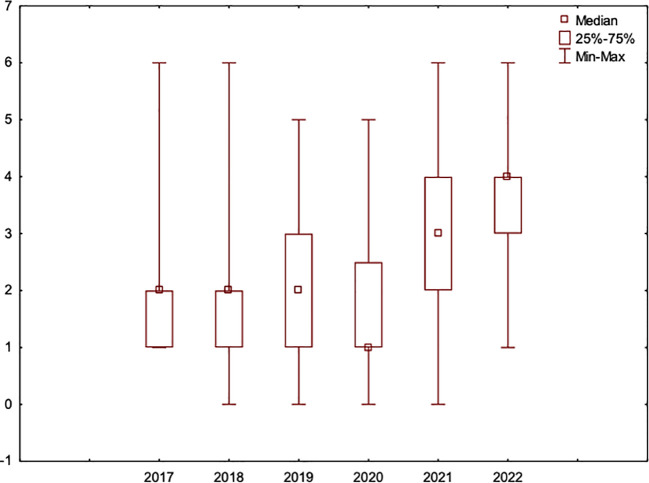
Younden Index indicating that 2020 was when the complication frequency trend changed. This moment coincides with the start of the regular prophylactic replacement procedure program.

The rate of TE fistula complications that occurred in the period of UVPR was 47% (425 out of 911 replacements), i.e., almost half of all replacement procedures resulted in complications when the procedure was unscheduled. After the introduction of PVPR in 2020, the rate of replacements with fistula complications in the first year alone significantly decreased to 15% (49 out of 329 replacements) and was the lowest in 2022 at 2% (12 out of 658). Throughout the entire PVPR period, the complication rate was 6% (91 out of 1,520 replacements). The statistical analysis showed that there was a correlation between an increased number of VP replacements and the incidence of complications per patient (r=0.339; p<0.001). Moreover, the Youden Index indicates that 2020 was the moment when the complication frequency trend decreased (AUC=0.812). This coincides with the beginning of the PVPR period (**Figure 1**). Logistic regression allowed us to determine that at the beginning of a follow-up, the chance of complications due to a VP exchange was twice as high [odds ratio (OR) = 1.990; 95% confidence interval (CI): 1.437–2.756; p < 0.001] compared to the period when cyclical VP exchange was introduced. In the following years when PVPR was utilized, the occurrence of complications systematically decreased in relation to the last year of the observation (OR = 0.026; 95% CI: 0.014–0.049; p < 0.001).

During the analyzed period, a marked decrease in the hospitalization length of patients due to complications related to TEF was observed following the implementation of PVPR. The total number of hospitalization days associated with VPR complications (TEF complications) was 1,820 days during the UVPR period and 436 during the PVPR period (r=0.387; p<0.001) (**Table 2**, **Table 4**). The difference between the total number of hospitalization days associated with complications and those related to uncomplicated VP exchanges (the latter of which, according to the NHS reimbursement requirement, must be performed within a 1-day hospitalization) was less pronounced. This was due to the increased number of VP exchanges during the PVPR period. Although the median number of hospitalization days per patient remained the same in both periods, i.e., 7.0 days, the total number of hospitalization days was higher during the UVPR period, totaling 2,731 days compared to 1,956 days in the PVPR period (**Table 3**).

It is particularly noteworthy that the number of individual patients who experienced complications was significantly reduced following the introduction of PVPR. In the UVPR period, complications occurred in 54% of all patients, compared to just 14% in the PVPR period (p<0.001).

**Figure 2:**
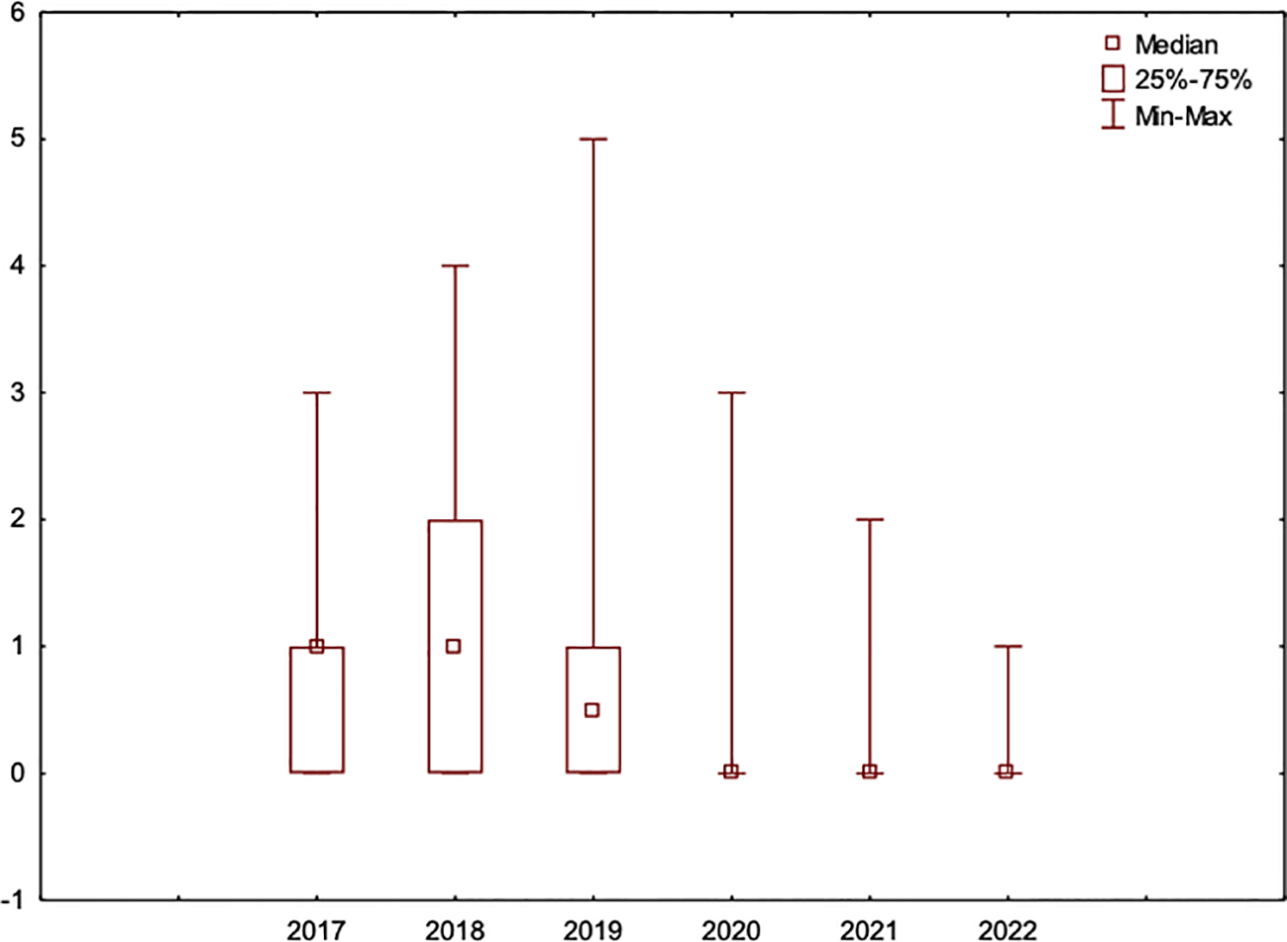
The number of VP replacements per patient per year in a group of 84 patients who were followed during the entire study period from 2017 to 2022.

**Figure 3:**
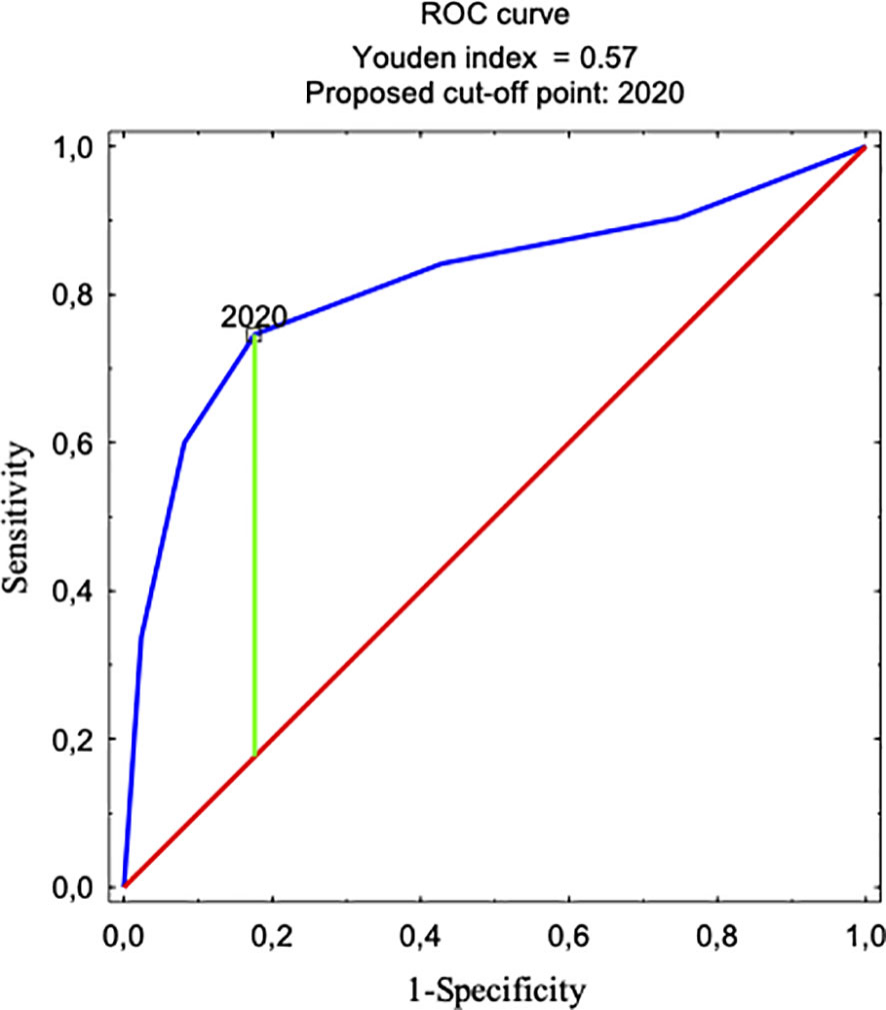
The number of complications per patient per year in a group of 84 patients who were followed during the entire study period from 2017 to 2022.

Moreover, 84 of all the included patients (n=327) were observed during the entire study period from 2017 to 2022. An additional analysis of this group was performed to confirm the trends found in the entire study group (**Figure 2**). The results also show that there was a statistically significant (p<0.001) lower ratio of complications in the PVPR period vs. the UVPR period in this smaller and homogenous group (**Figure 3**).

**Figure 4:**
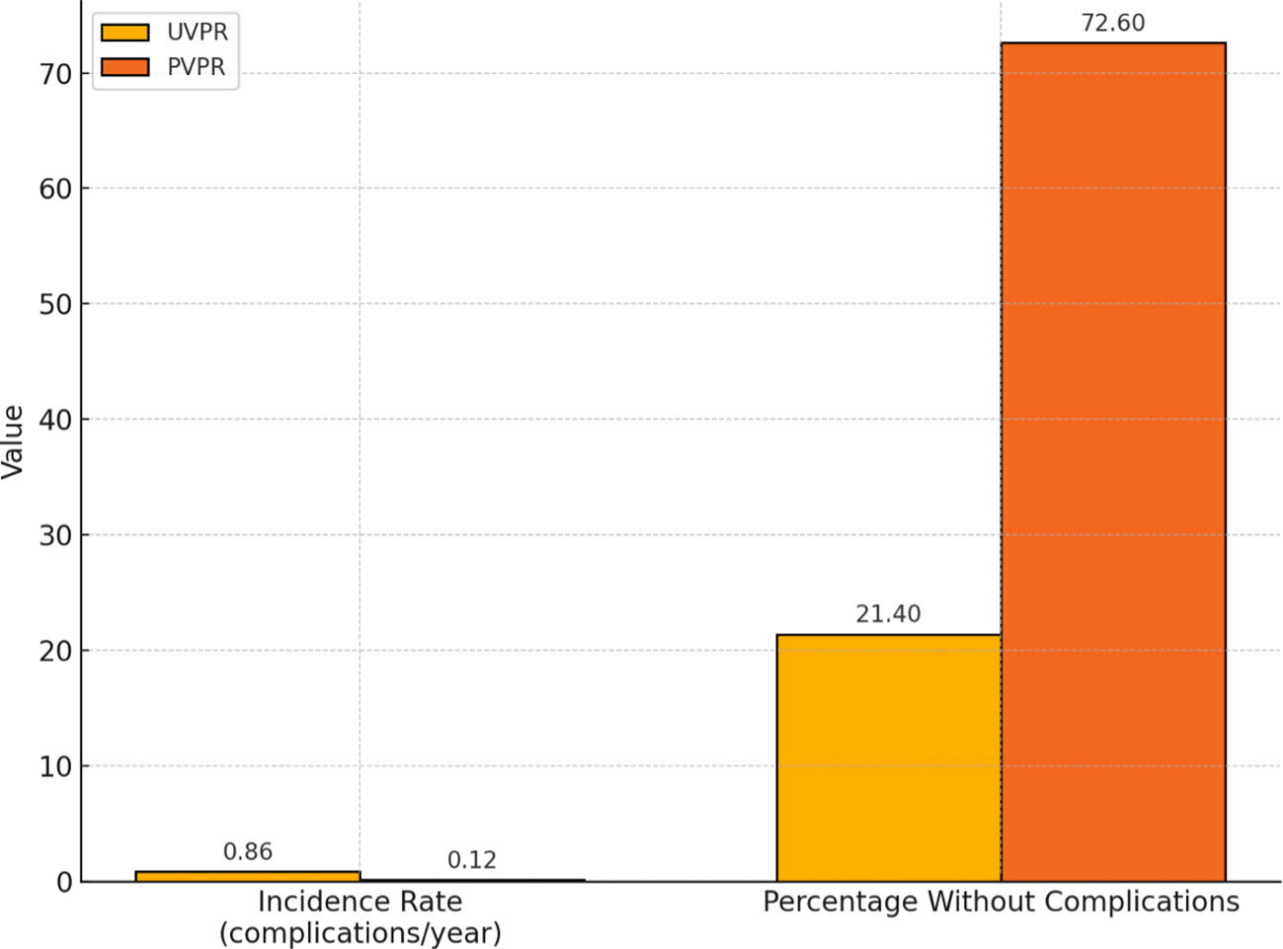
Comparison of the complication incidence rates and complication-free patient percentages between the UVPR and PVPR periods in the group of 84 patients. Note: The incidence rate of complications (expressed as complications per person-year) and the percentage of patients without any complications during the UVPR (2017–2019) and PVPR (2020–2022) periods were based on a dynamic cohort of 84 patients observed across both treatment strategies. A 3-year observation period was assumed for each patient in both the UVPR and PVPR phases.

Based on the analysis of the dynamic cohort of 84 patients, a significant reduction in the incidence of complications was observed after the introduction of the PVPR strategy (**Figure 4**). The mean incidence of complications decreased from 0.861 complications per person-year in the UVPR period (2017–2019) to 0.119 complications per person-year in the PVPR period (2020–2022). At the same time, the percentage of patients who remained completely free of complications during the given period increased from 21.4% in the UVPR to 72.6% in the PVPR. These results confirm the significant clinical benefit of the introduction of prophylactic, scheduled voice prosthesis replacement every 3 months.

Although the incidence of complications decreased in the PVPR period, the type of complications remained unchanged. The most frequent were TE fistula widening in 80% (n=425) and 78% (n=94) of cases in the PVPR and UVPR periods, respectively. No statistically significant difference in the type of complications was found between PVPR and UVPR. All the complications and the rate of their occurrence are presented in **Tables 5** and **6**.

**Table 5:** The occurrence of complications during VP replacement each year.

Type of complication	Occurrence of complications each year					
	2017 (n=130), n (%)*	2018 (n=165), n (%)*	2019 (n=130), n (%)*	2020 (n=49), n (%)*	2021 (n=33), n (%)*	2022 (n=12), n (%)*
TEF widening	109 (84)	117 (71)	115 (89)	39 (80)	25 (76)	9 (75)
TEF granulation	10 (8)	15 (9)	12 (9)	6 (12)	2 (6)	–
TEF inflammation	9 (7)	25 (15)	9 (9)	4 (8)	4 (12)	1 (8)
VP pulled into TEF	3 (2)	5 (3)	4 (3)	–	1 (3)	1 (8)
VP loss	19 (15)	11 (7)	15 (12)	6 (12)	1 (3)	4 (33)
Other and not specified	10 (8)	5 (3)	15 (12)	–	3 (9)	2 (17)

*The data do not add up to 100% because a patient may have experienced more than one type of complication. TEF, tracheoesophageal fistula; VP, voice prosthesis.

**Table 6:** The occurrence of complications during VP replacement in each period and over the total study period.

Type of complication	Total (2017-2022), n=519, n (%)*	UVPR (2017-2019), n=425, n (%)*	PVPR (2020-2022), n=94, n (%)*	p-value**
TEF widening	414 (80)	341 (80)	73 (78)	0.574
TEF granulation	45 (9)	37 (9)	8 (9)	0.951
TEF inflammation	52 (10)	43 (10)	9 (10)	0.874
VP pulled into TEF	14 (3)	12 (3)	2 (2)	0.706
VP loss	56 (11)	45 (11)	11 (12)	0.753
Other and not specified	35 (7)	30 (7)	5 (5)	0.543

*The data does not add up to 100% as a patient may have experienced more than one type of complication;** **Test χ2, p<α statistically significant; TEF, tracheoesophageal fistula; VP, voice prosthesis.

## 4 Discussion

The surgical method of voice and speech rehabilitation with VP implantation has become the gold standard and is increasingly used worldwide in patients after total laryngectomy. However, there are no accepted recommendations for the management/monitoring of patients with voice prostheses, even though this method may have some potentially life-threatening complications (7–9). Although there are papers proposing therapeutic procedures for the treatment of observed complications (11, 29), the work described here addresses the need for and examines the impact of preventing such complications. Reducing the number of complications and, therefore, increasing patient safety, is of paramount importance here. For several decades, there has been no significant technological progress in the field of VPs, both in terms of improved materials that are more resistant to biofilm and better-designed prostheses. The assessment of VP lifespan seems to be the most common topic in the literature on surgical voice rehabilitation, and is often combined with an evaluation of potential influencing factors. However, these assessments frequently lead to contrary conclusions. A prolonged life span of a correctly functioning VP is undoubtedly highly desirable from a patient’s perspective. However, from a medical standpoint, of unquestionable primary importance is patient safety, particularly concerning the occurrence of various complications associated with the use of VPs (8, 9, 12, 15, 21, 29–31, 34, 39). It is also challenging to compare studies on such complications associated with the use of VPs due to differences in methodology and the classification of observed complications. Often, these studies involve a small number of patients, reducing the significance of the observations.

The average device lifetimes reported in the literature range from 61 to 304 days (6, 15–28). In this study, the device lifetime in the UVPR period was 209 days, whereas in the PVPR period, it decreased to 133 days. Simultaneously, as the VP lifetime decreased, the number of complex prosthesis replacements due to TEF complications also significantly reduced, from 130 (41%) in 2017 to 12 (2%) in 2022. These complications affected 90 patients (52%) in 2017 and only 11 patients (6%) in 2022.

In 2020, a slight increase in the number of VP exchanges was observed, accompanied by a markedly lower incidence of those associated with complications and fewer days of extended hospitalizations. However, it should be noted that this year was unique due to the onset of the COVID-19 pandemic. Patients were reluctant to attend scheduled, planned voice prosthesis exchanges due to concerns about the risk of infection, coupled with more stringent hospital admission protocols being in place.

It should be noted that, compared to other publications, our retrospective single-center study has the highest number of patients with a proportionally shorter time of observation.

The main need for voice prosthesis replacement is due to device (VP) or fistula (TEF) related complications. Central leakage through the prosthesis is the most common complication, but the most demanding are periprosthetic leakage and TEP widening (11, 16, 30, 31), especially with atrophy of the fistula wall. In our observations, the incidence of all complications decreased in the PVPR period, but the type of complications remained unchanged. The most frequent was TE fistula widening, comprising 80% of all complication events in the PVPR period (n = 425) and 78% in the UVPR period (n = 94). No statistically significant difference in the type of complications was found between PVPR and UVPR. We presume that a prolonged DL and the consequent fungo-bacterial biofilm growth on the voice prosthesis may induce these fistula complications, however, a comparison of this study with others in literature is difficult as, thus far, there have been no publications strictly comparing the number of complications with the average duration of single prosthesis use. At the same time, the enlargement of the fistula and leakage around the prosthesis are the most frequent and the most challenging complications (11, 30–34). These observations are in line with our results that TEF enlargement with periprosthetic leakage was the most common and demanding complication (**Tables 5**, **6** **).**

There are studies by other authors that suggest some factors that influence device lifetime and the complication rate, however, there are many discrepancies due to the use of different methodologies and small groups of observed patients. Pre- and postoperative radiotherapy is considered the most significant factor in decreasing device lifetime and increasing the number of complications in laryngectomized patients with the VPs (3, 35–38), but there are papers that contradict this (11, 39). Another factor that is considered a negative is gastroesophageal reflux disease (GERD). Patients with GERD often have a higher risk of TEF enlargement and shorter voice prosthesis lifespan (37, 40–42). Margolin et al. found there was a significant correlation between the occurrence of TEF complications and the severity of reflux (42).

There are also many other proposed reasons for complications related to TEF in the literature, which often contradict each other. Most of these studies are based on small patient groups and different methodologies. Therefore, it seems there is a need to create a unified scheme for evaluating complications that can be used by clinicians in various centers to objectively determine the most important factors affecting both the lifespan and, most importantly, the occurrence of complications related to a tracheoesophageal fistula. Based on this, recommendations for an optimal follow-up strategy for patients with VPs could be developed.

We postulate that a shorter voice prosthesis lifetime (3 months) is associated with a significantly decreased risk of TE fistula complications. It is very likely that increased biofilm formation on the VP acts as an irritant to the fistula tract and eventually can lead to widening, infection, or granulation of the local tissue. The presence of mainly *Candida* fungo-bacterial biofilm on the VP and deterioration of the silicon material of the VP that worsens over time have been confirmed in previous studies (13, 43–46).

The benefits of prophylactic prosthesis replacement do not only concern shortening the time of its functioning and consequently, the amount of biofilm, but also better organization of the clinic’s work, avoiding sudden unplanned admissions/replacements seems to be an equally important benefit that significantly improves the patient’s safety. Planned replacements mean that such a procedure is performed by the most qualified personnel who have enough time to properly perform the voice prosthesis replacement. Some of these complications can be caused by an incorrect replacement procedure traumatizing the fistula wall, incorrect prosthesis size, the lack of sufficient experience, combined with fear of complications that could lead to unnecessarily prolonged hospitalization, as well as the limited availability of the doctor to whom the patient with voice prosthesis dysfunction was referred, also contributed to the issue. During a planned voice prosthesis replacement visit, there is also time for proper oncological control of patients who are exposed not only to a possible recurrence of the laryngeal cancer, but also to other primary lesions. Even though it is beneficial for both patients and medical staff, some patients still refuse PVPR if their device works properly.

It is worth noting that, in some countries, the number of VPs used per patient is already higher than the number that we were trying to reach in this study (four per year).

The economic aspect of healthcare should not be overlooked (**Table 7**). During the UVPR period, 911 voice prostheses (350 euros per VP) were utilized, along with 911 1-day standard hospitalizations for the VPR procedure (275 euros per day), resulting in a total cost of 569,375 euros. Additionally, there were 1,820 extra days of hospitalization due to complications, amounting to a cost of 500,500 euros. Therefore, the total cost for the UVPR period was 1,069,875 euros. In the PVPR period, 1,520 voice prostheses (350 euros per VP) were used, along with 1,520 one-day standard hospitalizations for the VPR procedure (275 euros per day), totaling 950,000 euros. With only 436 extra days of hospitalization due to complications, the cost was 119,900 euros. The total cost for the PVPR period was 1,069,900 euros. Based on these calculations, PVPR appears to be cost-neutral from a healthcare provider perspective and offers clear benefits for the patients. Of note, the median number of hospitalization days per patient was the same in the UVPR and PVPR periods. However, patients with complications required more expensive treatment than those hospitalized solely for scheduled VPR procedures. These calculations pertain to prosthesis exchanges and treatment of related adverse effects within a hospital setting, however, the outpatient treatment option also carries costs related to the management of complications, some of which are borne by the patients themselves. Furthermore, the costs associated with medical leave may be impactful for working patients.

**Table 7:** Calculation of the costs of the UVPR and PVPR policies.

Expenses	UVPR	PVPR
Total number of VP replacement procedures	911	1520
Cost of all VPs (350 euro per single VP)	318–850 euro	532–000 euro
Cost of all 1-day hospitalizations for VP replacement (275 euro per day of hospitalization)	250–525 euro	418–000 euro
Total extra days of hospitalization due to fistula complications	1820	436
Cost of all 1-day hospitalizations for VP replacement (275 euro per day of hospitalization)	500–500 euro	119–900 euro
**TOTAL COST**	**1 069–875 euro**	**1 069–900 euro**

VPR, voice prosthesis replacement; UVPR, unscheduled voice prosthesis replacement; PVPR, prophylactic voice prosthesis replacement.

In 2023, Heirman et al. published a study on prophylactic voice prosthesis replacement based on a theoretical model, with the conclusion that it is not feasible (47). Their aim was to prevent patients from unexpected leakages associated with the proper function of the voice prosthesis, not with TE fistula complications. The median device lifetime in their study group was approximately 2 months, far shorter than the device lifetime we found at the end of the PVPR period.

### 4.1 Limitations

In this study, we analyzed all types of complications. Every time a patient was hospitalized for more than 1 day, it was considered a complication. When a patient was hospitalized for only 1 day, it was considered a routine replacement without any complications. It is worth noting that the NHS regulations in Poland require voice prosthesis replacement to be a hospital procedure; thus, admitting the patient for at least 1 day was and still is an obligation. In cases with fistula inflammation, granulation, or widening, the voice prosthesis was removed from the fistula, and insertion of the new one was delayed until the TE tissue healed, with the patients remaining in the hospital until then. The management of fistula problems and the indications for admitting patients to the hospital are different worldwide. In many countries, most fistula problems are handled in an outpatient clinic.

Human factors may also play a role in fistula complication assessment. The lower number of complications during the PVPR period may have been due to more experienced physicians who performed voice prosthesis replacements in a more organized, planned manner during this period.

## 5 Conclusions

Prophylactic voice prosthesis replacement every 3 months reduced fistula complications compared to a protocol involving reactive replacement due to voice prosthesis or tracheoesophageal fistula dysfunction. This approach also led to a decrease in the number of emergency visits and enhanced patient safety, while simultaneously reducing the number of patients who experienced related complications. The types of complications remained unchanged, with tracheoesophageal fistula widening being the most frequent. Notably, PVPR was found to be cost-neutral in our setting.

## Data Availability

The raw data supporting the conclusions of this article will be made available by the authors, without undue reservation.
